# Loss of Free Fatty Acid Receptor 2 leads to impaired islet mass and beta cell survival

**DOI:** 10.1038/srep28159

**Published:** 2016-06-21

**Authors:** Stephanie R. Villa, Medha Priyadarshini, Miles H. Fuller, Tanya Bhardwaj, Michael R. Brodsky, Anthony R. Angueira, Rockann E. Mosser, Bethany A. Carboneau, Sarah A. Tersey, Helena Mancebo, Annette Gilchrist, Raghavendra G. Mirmira, Maureen Gannon, Brian T. Layden

**Affiliations:** 1 Division of Endocrinology, Metabolism and Molecular Medicine, Northwestern University Feinberg School of Medicine, Chicago, IL, USA; 2 Vanderbilt University, Department of Medicine, Division of Diabetes, Endocrinology and Metabolism, Nashville, TN, USA; 3 Vanderbilt University, Department of Molecular Physiology and Biophysics, Nashville, TN, USA; 4 Department of Pediatrics and the Herman B Wells Center for Pediatric Research, Indiana University School of Medicine, Indianapolis, IN, USA; 5 Multispan Inc., Hayward, CA, USA; 6 Midwestern University Department of Pharmaceutical Sciences, Downers Grove, IL, USA; 7 Department of Biochemistry and Molecular Biology, Indiana University School of Medicine, Indianapolis, IN, USA; 8 Department of Medicine, Indiana University School of Medicine, Indiana University, Indianapolis, IN, USA; 9 Tennessee Valley Health Authority, Department of Veterans Affairs, Nashville, TN, USA; 10 Jesse Brown Veterans Affairs Medical Center, Chicago, IL, USA

## Abstract

The regulation of pancreatic β cell mass is a critical factor to help maintain normoglycemia during insulin resistance. Nutrient-sensing G protein-coupled receptors (GPCR) contribute to aspects of β cell function, including regulation of β cell mass. Nutrients such as free fatty acids (FFAs) contribute to precise regulation of β cell mass by signaling through cognate GPCRs, and considerable evidence suggests that circulating FFAs promote β cell expansion by direct and indirect mechanisms. Free Fatty Acid Receptor 2 (FFA2) is a β cell-expressed GPCR that is activated by short chain fatty acids, particularly acetate. Recent studies of FFA2 suggest that it may act as a regulator of β cell function. Here, we set out to explore what role FFA2 may play in regulation of β cell mass. Interestingly, *Ffar2*^−/−^ mice exhibit diminished β cell mass at birth and throughout adulthood, and increased β cell death at adolescent time points, suggesting a role for FFA2 in establishment and maintenance of β cell mass. Additionally, activation of FFA2 with Gα_q/11_-biased agonists substantially increased β cell proliferation in *in vitro* and *ex vivo* proliferation assays. Collectively, these data suggest that FFA2 may be a novel therapeutic target to stimulate β cell growth and proliferation.

In response to states of chronic insulin resistance, pancreatic islets employ multiple compensatory responses in an attempt to maintain whole-body glucose homeostasis. These responses consist of enhanced insulin secretion as well as expansion of beta (β) cell mass^1^. When an individual is unable to sustain these compensatory mechanisms, due to a confluence of genetic, environmental, and/or lifestyle factors, progression to Type 2 diabetes (T2D) can occur. The specific contribution of β cell mass deficits versus impaired β cell function in the progression to *de facto* T2D remains a matter of some debate^2^. However, several lines of evidence suggest that initial β cell loss (possibly occurring as early as the pre-diabetic phase) places increased secretory burden on the surviving β cells, leading to chronic β cell stress and further impairments in β cell function as a result of β cell exhaustion^3^,^4^,^5^,^6^. Along these lines, impaired pre- or postnatal development of β cells is suggested to predispose some individuals to T2D when exposed to aggravating factors such as obesity and insulin resistance^2^. This possibility is illustrated by studies in which factors such as genetic polymorphisms and fetal malnutrition have been shown to impair β cell mass and result in increased diabetes risk later in life^7^,^8^,^9^,^10^.

Consistent with these reports, the nutritional status of an individual is thought to be an important regulator of β cell mass. Multiple nutrients such as glucose, amino acids, and free fatty acids contribute to maintain precise regulation of β cell mass^11^. For example, evidence suggests that circulating levels of glucose and free fatty acids (FFA) can promote β cell expansion, although this remains a matter of some debate^12^. Additionally, nutrient sensing via the gut may indirectly contribute to regulation of β cell mass by promoting GLP-1 secretion from intestinal L-cells, which in turn acts at the β cell to promote cell survival and proliferation. Many nutrients and nutrient-regulated factors exert their influence through G protein-coupled receptors (GPCRs), consistent with the well characterized ability of these receptors to regulate multiple aspects of β cell function and health, including glucose-stimulated insulin secretion (GSIS) and β cell survival and proliferation^13^. For example, chronic signaling through Gα_q/11_ and Gα_s_ by designer GPCRs enhanced β cell mass as a result of increased β cell proliferation and β cell hypertrophy^14^,^15^. In support of this observation, signaling via the Gα_s_-coupled GLP-1 receptor by the agonist Exendin-4 improves β cell function, potentiating GSIS and enhancing β cell replication and neogenesis^16^. Similarly, activation of Gα_q/11_-coupled receptors such as the M3 muscarinic and long chain free fatty acid receptor FFA1 potentiate GSIS and have been suggested to promote β cell survival and proliferation^13^,^17^,^18^. In contrast, activation of Gα_i/o_^19^,^20^ or Gα_z_^21^ pathways inhibits β cell function and proliferation.

In addition to FFA1, multiple other FFA-sensing GPCRs have been identified in the β cell, and have garnered considerable interest as potential targets for the treatment of T2D in recent years^22^,^23^. Recently, our group and others have reported that islet expression of the short chain fatty acid receptor FFA2 is dynamically regulated in association with multiple models of insulin resistance, including pregnancy and diet-induced and genetic models of obesity and diabetes^24^,^25^,^26^. The endogenous ligands of FFA2, short chain fatty acids, are derived primarily from fermentation of dietary fiber by gut flora^27^, positioning FFA2 as one possible link between the gut microbiome and its host. These observations have led us to explore and describe a role for FFA2 in affecting crucial aspects of β cell biology. These studies revealed that FFA2 signaling can either stimulate GSIS via the Gα_q/11_ pathway or inhibit GSIS via Gα_i/o_^28^. Specifically, we found that two different classes of previously described FFA2 agonists, small carboxylic acids and phenylacetamide derivatives, demonstrated a bias toward activating Gα_q/11_ or Gα_i/o_ pathways, respectively. Around the same time, two other groups published their findings, with McNelis *et al.* reporting dual coupling of FFA2 to Gα_i/o_ and Gα_q/11,_ with potentiation of GSIS mediated primarily by Gα_q/11_^29^. By contrast, Tang *et al.*, observed solely Gα_i/o_ signaling via FFA2, leading to inhibition of GSIS^30^. With respect to β cell mass, Tang *et al.*, and McNelis *et al.* also reported conflicting findings. Whereas Tang *et al.* reported no differences in islet morphology between WT and *Ffar2*^−/−^ mice^30^, McNelis *et al.* reported a defect in β cell mass expansion in response to insulin resistance as a result of *Ffar2* deletion^29^. Because of the importance of β cell mass in T2D pathogenesis, here we sought to clarify a potential role for FFA2 in regulating β cell mass and proliferation, and to determine how biased signaling of FFA2 through Gα_q/11_ or Gα_i/o_ may determine its effect on β cell mass.

## Results

### Genetic deletion of *Ffar2* impairs postnatal β cell mass

**Figure 1:**
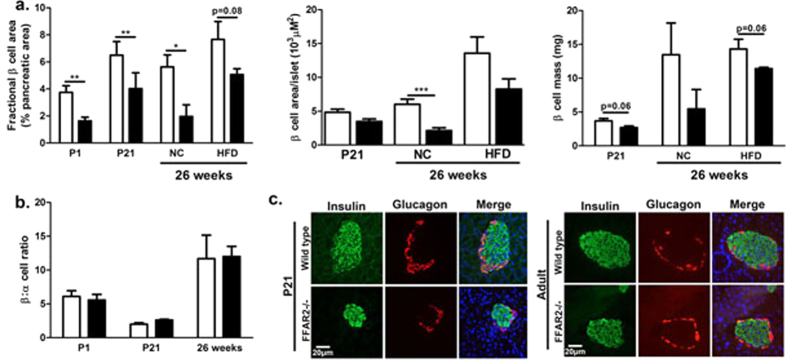
Genetic deletion of *Ffar2* impairs postnatal β cell mass. (**a**) β cell area, average β cell area per islet, and β cell mass of WT and *Ffar2*^−/−^ mice. Adult mice were fed either normal chow (NC) or high fat diet (HFD) as indicated; β cell area is expressed as percent insulin positive area relative to total pancreatic area. (**b**) β to α cell ratio at each indicated time point. (**c**) Representative images of WT and *Ffar2*^−/−^ islets at P21 and 26 weeks. Scale bar = 20 μm. Green: insulin, red: glucagon. For all experiments, n ≥ 3; white bars = WT, black bars = *Ffar2*^−/−^. *p < 0.05, **p < 0.01 and ***p < 0.001.

In T2D, the loss of functional β cell mass is an important factor in disease-development^31^. As GPCRs have been described to contribute to β cell mass through their role in mediating β cell properties such as proliferation and apoptosis^13^,^14^,^15^,^16^,^17^,^18^,^19^,^20^, we examined the effect of *Ffar2* deletion on β cell mass. Immunohistochemical (IHC) and morphometric analysis of normal chow (NC) fed *Ffar2*^−/−^ male mice at 26 weeks revealed significantly decreased insulin positive area (relative to total pancreatic area), smaller average β cell area per islet, and decreased β cell mass compared to age matched, WT controls (Fig. 1a). Notably, we have also observed this phenotype in a separate study of 10–12 week old *Ffar2*^−/−^ females^32^. To examine the influence of FFA2 in regulating adaptive β cell mass expansion in response to insulin resistance, we also assessed β cell mass in WT and *Ffar2*^−/−^ mice at 26 weeks, following 20 weeks of high fat diet (HFD). Although the overall trend toward diminished β cell mass persisted in HFD-fed *Ffar2*^−/−^ mice, β cell mass in *Ffar2*^−/−^ islets did increase in response to HFD, suggesting that mice with deletion of *Ffar2* retain some capacity for adaptive β cell mass expansion in response to HFD (Fig. 1a).

Given the diminished β cell mass in adult *Ffar2*^−/−^ mice under normal diet conditions, we next examined β cell mass at younger ages, specifically, immediately postnatal (P1) and at 21 days (P21), to determine when the β cell mass phenotype became apparent. Interestingly, we observed significant reductions in the β cell area of *Ffar2*^−/−^ mice at both ages, and marginally decreased β cell mass at P21, suggesting that impairments in β cell mass develop during the prenatal period in *Ffar2*^−/−^ mice (Fig. 1a). Despite these impairments, overall islet architecture and α to β cell ratio was unaffected at each age examined (Fig. 1b,c).

### *Ffar2^−/−^* islets demonstrate increased β cell death *in vivo*

**Figure 2:**
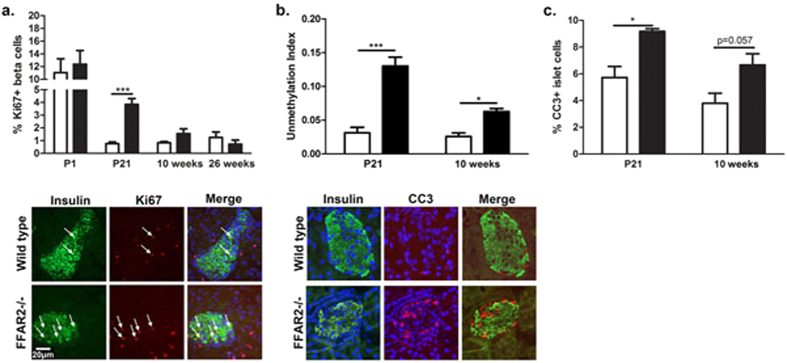
*Ffar2*^−/−^ islets do not demonstrate impaired proliferation, but have increased β cell death. (**a**) β cell proliferation in WT and *Ffar2*^−/−^ mice as determined by Ki67 co-localization with insulin-positive cells at P1, P21, 10 weeks, and 26 weeks. Bottom: Representative images at P21 shown, scale bar = 20 μm. Green: insulin, red: Ki67. (**b**) Preproinsulin unmethylation index for WT and *Ffar2*^−/−^ mice at indicated time points. (**c**) β cell apoptosis in WT and *Ffar2*^−/−^ mice as determined by cleaved caspase 3 (CC3) staining at P21 and 10 weeks. Bottom: Representative images at P21 shown. For all experiments, n ≥ 3; white bars = WT, black bars = *Ffar2*^−/−^. *p < 0.05, **p < 0.01 and ***p < 0.001.

To better understand the mechanism by which *Ffar2* contributes to β cell mass, we next assessed β cell proliferation, as this is a major factor regulating postnatal β cell mass, and is modulated in part by GPCR signaling^33^. Using Ki67 immunostaining to assess β cell proliferation at early-postnatal (P1), adolescent (P21), and adult (10 weeks and 26 weeks) ages, we observed dramatically increased β cell proliferation in *Ffar2*^−/−^ pancreata at P21 and a trend toward increased proliferation at 10 weeks, but found no differences in β cell proliferation between WT and *Ffar2*^−/−^ mice at P1 or 26 weeks on NC diet (Fig. 2a). Thus, these data do not suggest that impaired β cell proliferation is a primary mechanism leading to diminished β cell mass in *Ffar2*^−/−^ pancreata.

Given these findings, we suspected that diminished β cell mass may stem from increased rates of β cell death in *Ffar2*^−/−^ mice. Recent studies have reported that β cell death results in an increase in serum unmethylated preproinsulin DNA, the presence of which may serve as a reliable biomarker of β cell death^34^,^35^,^36^,^37^. We therefore analyzed the serum of WT and *Ffar2*^−/−^ mice at P21 and 10 weeks of age by multiplex PCR and found a significantly higher unmethylation index in *Ffar2*^−/−^ mice at both time points, indicating increased β cell death in *Ffar2*^−/−^ mice (Fig. 2b); these findings were further supported by observations of increased cleaved caspase 3 staining in *Ffar2*^−/−^ islets at P21 and 10 weeks (Fig. 2c). Taken together, these data suggest that *Ffar2* contributes to the prenatal establishment of β cell mass, and that deletion of *Ffar2* results in increased postnatal β cell death, suggesting a role for FFA2 in supporting β cell survival.

### Acetate does not promote FFA2 mediated β cell proliferation

**Figure 3:**
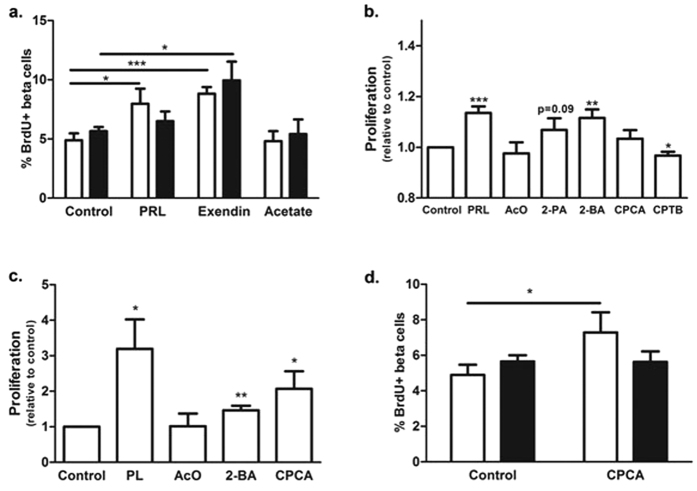
Biased agonism of FFA2 promotes β cell proliferation. (**a**) β cell proliferation in intact islets from WT and *Ffar2*^−/−^ mice following treatment with prolactin (PRL, 0.5 μg/ml), Exendin-4 (100 nM), and acetate (1 mM). (**b**) β cell proliferation in INS1 cells following treatment with prolactin (PRL, 0.5 μg/ml), acetate (AcO, 1 mM), 2-propynoic acid (2-PA, 0.5 mM), 2-butynoic acid (2-BA, 5 mM), cyclopent-1-enecarboxylic acid (CPCA, 0.5 mM), or CPTB (5 μM), as determined by flow cytometry. (**c**) β cell proliferation in intact WT islets following treatment with placental lactogen (PL, 0.5 μg/ml), acetate (AcO, 1 mM), 2-butynoic acid (2-BA, 5 mM), or cyclopent-1-enecarboxylic acid (CPCA, 0.5 mM). (**d**) β cell proliferation in intact islets from WT and *Ffar2*^−/−^ mice following treatment with CPCA (0.5 mM). For all experiments, n = 3. *p < 0.05, **p < 0.01 and ***p < 0.001. For **a** and **d**, white bars represent WT, black barsrepresent *Ffar2^−/−^.*

As Gα_q/11_-coupled GPCRs are well known to mediate β cell proliferation^15^, we more directly examined whether FFA2 signaling can influence β cell proliferation using *ex vivo* studies with intact mouse islets from 10–12 week old WT and *Ffar2*^−/−^ mice. Here, we did not observe any significant differences in the rate of proliferation between WT and *Ffar2*^−/−^ β cells under untreated conditions, nor in response to the primary endogenous FFA2 ligand, acetate or the GLP-1 receptor agonist, exendin 4, which is known to potently enhance β cell proliferation (Fig. 3a). It is noteworthy, however, that treatment with prolactin, which is also known to stimulate β cell proliferation, did not significantly enhance proliferation in *Ffar2*^−/−^ β cells (Fig. 3a)^16^,^38^. This observation is especially important in light of our recent findings that adaptive β cell mass expansion during pregnancy is compromised in *Ffar2*^−/−^ female mice^32^, as this adaptive expansion is regulated in large part by prolactin receptor (PRLR) signaling^39^. Moreover, a gene expression analysis by our group revealed no difference in PRLR expression in WT and *Ffar2*^−/−^ islets or commonly associated downstream genes to the PRLR pathway (Supplementary Table 1); thus, the impaired proliferative response to prolactin does not appear to result from impaired gene expression in the PRLR pathway. However, these findings suggest that while there are no apparent differences in proliferation between WT and *Ffar2*^−/−^ β cells under basal conditions, *Ffar2*^−/−^ β cells may be impaired in their capacity to respond to some proliferative factors, and further investigation into the relationship between FFA2 and PRLR signaling is needed.

### Gα_q/11_-biased agonism of FFA2 promotes β cell proliferation

As noted above, acetate did not appear to influence β cell proliferation. However, we have previously demonstrated that some FFA2-specific agonists demonstrate biased G-protein signaling at mouse FFA2 via either Gα_q/11_ or Gα_i/o_ pathways^28^. Because FFA2 couples to both Gα_q/11_ and Gα_i/o_, which have been described to either enhance or inhibit β cell proliferation^15^,^20^, respectively, we next explored the effect of biased Gα_q/11_ or Gα_i/o_ signaling via FFA2 on β cell proliferation. Assessing the effect of Gα_q/11_- and Gα_i/o_-biased FFA2 agonists on β cell proliferation *in vitro* in the INS1 rat β cell line, we found that 48 hours treatment with the Gα_q/11_-biased agonist 2-butynoic acid (2-BA, previously referred to as SCA15), elicited a significant increase in β cell proliferation as determined by BrdU incorporation (Fig. 3b). Further, another Gα_q/11_-biased agonist, 2-propynoic acid (2-PA, previously referred to as SCA14), elicited a considerable, but non-significant increase in β cell proliferation (Fig. 3b). Conversely, treatment with the Gα_i/o_-biased agonist CPTB inhibited β cell proliferation, consistent with a model of Gα_q/11_-mediated potentiation of β cell proliferation and Gα_i/o_-mediated inhibition of β cell proliferation *via* FFA2.

To test these observations in a more physiologically relevant model, we conducted an initial screening of the Gα_q/11_-biased compounds in isolated, intact WT islets, and proliferation was assessed by BrdU incorporation. Here we found that treatment with 5 mM 2-BA and 0.5 mM cyclopent-1-enecarboxylic acid (CPCA) resulted in a substantial increase in β cell proliferation relative to untreated islets (Fig. 3c), while 2-PA, which promoted proliferation in INS1 β cells, had no effect in mouse islets (data not shown). Thus, we next tested CPCA, which elicited the largest increase in proliferation in our initial islet studies, in WT and *Ffar2*^−/−^ islets, and observed a 49% increase in proliferation in WT, but not *Ffar2*^−/−^ islets, demonstrating that this effect is in fact FFA2-dependent (Fig. 3d). We further explored the signaling pathways activated by CPCA using *in vitro* calcium mobilization and cAMP accumulation assays, which are indicators of Gα_q/11_ and Gα_i/o_ signaling pathways, respectively. Here, CPCA demonstrated relatively potent calcium mobilization in the BTC3 β cell line, but less potency in the cAMP assay, consistent with Gα_q/11_-biased signaling (Table 1). These data are compared to acetate, which does not affect β cell proliferation, and demonstrates higher potency for cAMP inhibition and less potent calcium mobilization. Taken together, these data suggest that Gα_q/11_-biased stimulation of FFA2 by CPCA can promote β cell proliferation.

## Discussion

Growing evidence that the loss of functional β cell mass is a primary mechanism of T2D pathogenesis suggests that the identification of novel targets that promote β cell growth, proliferation, and survival may be especially desirable targets for early disease intervention. Thus, here we assessed the role of FFA2 in regulating β cell mass. We observed impaired β cell mass at birth, and throughout adulthood in *Ffar2*^−/−^ mice. *In vitro* assays of β cell proliferation in WT and *Ffar2*^−/−^ islets did not reveal any intrinsic differences in proliferation at baseline in *Ffar2*^−/−^ β cells, nor in their response to the GLP1 receptor agonist Exendin 4, but our data did suggest that *Ffar2*^−/−^ islets may be compromised in their ability to respond to prolactin. This raises the possibility that *Ffar2*^−/−^ mice may experience a developmental impairment arising from decreased sensitivity to developmental signaling factors. Interestingly, we have previously reported impaired β cell mass expansion and glucose tolerance in *Ffar2*^−/−^ female mice during pregnancy. As prolactin is a major driver of β cell mass expansion during pregnancy^39^,^40^, these data further point to the relationship between PRLR and FFA2 signaling as an important future avenue of inquiry.

Surprisingly, at 21 days of age, *Ffar2*^−/−^ mice exhibit increased β cell proliferation, which appears to be secondary to a large increase in β cell death, which is detectable until at least 10 weeks of age. These observations suggest that FFA2 also contributes to supporting β cell survival during adolescence and early adulthood. It is noteworthy that despite our observations of diminished β cell mass in *Ffar2*^−/−^ mice, we have previously reported that these mice do not exhibit any overt metabolic phenotype *in vivo*^28^. However, we have observed that isolated *Ffar2*^−/−^ islets secrete significantly less insulin than WT islets in response to stimuli such as high glucose and the GLP-1 receptor agonist, Exendin-4^28^. Taken together, these data suggest that in our mouse model, sufficient compensation occurs to maintain overall glucose homeostasis in *Ffar2*^−/−^ mice, despite impaired GSIS *in vitro* and diminished β cell mass.

Notably, two other groups have recently published their findings regarding the effect of *Ffar2* deletion on β cell function^29^,^30^. While Tang *et al.*^30^ reported no morphological differences in the islets of their WT and *Ffar2*^−/−^ mice, it is significant that they did not conduct *de facto* β cell mass or area analysis. More significantly, McNelis *et al.*^29^, did not identify any impairments in β cell area from *Ffar2*^−/−^ mice under normal diet conditions, although they did observe impaired adaptive β cell mass expansion in *Ffar2*^−/−^ mice on HFD, along with impaired glucose tolerance. In their mouse model, these impairments stemmed from a failure of *Ffar2*^−/−^ β cells to proliferate. In our studies, we observed a notable, but not significant defect in β cell proliferation following 1 week HFD or 20 weeks of HFD in *Ffar2*^−/−^ mice (Supplementary Fig. 1). However, our analyses were conducted very early and very late in insulin resistance (1 week and 20 weeks, respectively), and may have missed the window of maximal compensatory β cell proliferation. Moreover, it has been noted that the phenotype elicited by HFD can vary greatly, depending on factors such as the genetic background, the diet’s micro- and macronutrient composition, and the timing of high fat-feeding^41^.

Because expansion of β cell mass is a critical component of islet adaptation to insulin resistance and the maintenance of glucose homeostasis, methods to promote β cell growth and proliferation offer invaluable therapeutic tools for T2D. As we previously reported, some FFA2 agonists demonstrate signaling bias, preferentially signaling through Gα_i/o_ or Gα_q/11_, which results in differential effects on GSIS^28^. Likewise, our data here suggest that these differential effects may extend to the regulation of β cell proliferation. Encouragingly, our studies suggest that FFA2 may be a novel target to promote β cell proliferation and survival. As the *in vivo* metabolism and toxicity of these FFA2 agonists is unknown, the compounds are currently unsuitable for additional experimentation, thus, additional work is underway to develop new Gα_q/11_-biased FFA2 agonists that are more suitable for *in vivo* use. However, *in vitro* and *ex vivo* testing of these agonists have demonstrated the potential of Gα_q/11_-biased FFA2 to substantially increase β cell proliferation in intact islets. These studies are in agreement with the recent report by McNelis *et al.*, who observed a statistically significant increase in β cell proliferation following treatment with the phenylacetamide agonist PA1, which they reported to be primarily Gα_q/11_-mediated. Taken together, these two independent studies point to FFA2 as a potential target to promote β cell proliferation.

It is especially noteworthy that we have previously observed PA1 (referred to herein as CPTB), to be primarily Gα_i/o_-biased, while the publication by McNelis *et al.*, suggests a Gα_q/11_-biased signaling mechanism. Accordingly, while McNelis *et al.* report PA1-mediated increases in β cell proliferation and insulin secretion, we have observed PA1-mediated inhibition of β cell proliferation and insulin secretion. As discussed in recent studies of FFA2^28^,^29^,^30^,^42^,^43^, these discrepancies may result from the use of different mouse models made by different methods and on different backgrounds. Other factors may contribute to the widely varying phenotypes observed across multiple studies. Specifically, GPCR coupling can be regulated by numerous factors, for example by dimerization with other receptors^44^ or interaction with modifying proteins^45^, both of which may be modulated by the physiologic state of the host. Given the apparent complexity of FFA2 signaling and the *Ffar2*^−/−^ phenotype, it is critical that we develop a deeper understanding of how FFA2 signaling and coupling is regulated. Considering FFA2’s role as a nutrient sensor, future studies aimed at understanding what factors underlie the regulation of FFA2 coupling and signaling in different physiologic and biochemical states will be of particular importance in helping to guide the development of FFA2 into a viable therapeutic target for T2D.

## Methods

### Animals

Heterozygous *Ffar2* knockout mice (*Ffar2*^+/−^) on a C57BL/6J background were crossed to produce *Ffar2* wild-type (WT) and *Ffar2* knockout (*Ffar2*^−/−^) mice. Genotypes were determined as previously described^42^. Mice were housed in a temperature-controlled facility with 12-hour light-dark cycle, and given *ad libitum* access to water and normal (LM-485, Harlan Laboratories, Indianapolis, IN) or high fat chow (TD.06414, Harlan Laboratories, started at 6 or 10 weeks of age). All experiments described in this report were carried out in accordance with protocols approved by the Institutional Animal Care and Use Committee at Northwestern University.

### Tissue processing, immunolabeling, and morphometric analysis

Mouse pancreata were immediately removed from euthanized mice, weighed, and fixed in 4% paraformaldehyde in 1X PBS for 1 hour and kept in 30% sucrose in PBS overnight. The next day, pancreata were cryopreserved by embedding in optimal-cutting-temperature compound and stored at −80 °C until further processing. Pancreata were sectioned at 7 μm using a microtome-cryostat. For morphometric analysis, three to four non-overlapping sections from each pancreas (spanning the width of the pancreas) were used for each analysis. All analyses were conducted on at least 3 animals per age, genotype, and treatment condition.

For determination of β cell mass, sections were immunolabeled with guinea pig anti-insulin and rabbit anti-glucagon primary antibodies and imaged by epifluorescence at 4X and 40X magnification. The total pancreatic area and insulin positive area of each section was measured using ImageJ software (http://imagej.nih.gov/ij/). β cell mass was estimated as the product of the relative β cell area and the wet weight of the pancreas.

β cell proliferation was examined by immunolabeling with guinea pig anti-insulin and rabbit anti-Ki67 primary antibodies. Islets were imaged at 40X, and the number of insulin-positive and insulin- and Ki67-positive cells counted, and proliferation was calculated as the percentage of Ki67-positive β cells.

β cell apoptosis was examined by immunolabeling with guinea pig anti-insulin and rabbit anti-cleaved caspase 3 primary antibodies. Islets were imaged at 40X, and the number of insulin-positive cells counted. Cleaved caspase 3-positive cells were counted as apoptotic islet cells if they co-localized with insulin, or were located within the insulin-positive islet area.

The following primary antibodies were used, all diluted in antibody diluent buffer (Dako, Carpinteria, CA): guinea pig anti-insulin (1:200; Abcam, Cambridge, MA), rabbit anti-glucagon (1:200; Cell Signaling, Danvers, MA), rabbit anti-Ki67 (1:100; Cell Signaling), rabbit anti-cleaved caspase 3 (1:2000, Abcam, Cambridge, MA). Goat-derived secondary antibodies conjugated to Fluorescein or Texas Red were used at 1:200 dilution (Vector Laboratories, Burlingame, CA). All slides were mounted with Vectashield with DAPI (Vector Laboratories).

### Islet isolation and culture

Islets were isolated by collagenase digestion as previously described^28^. Following isolation, islets were handpicked under a dissection microscope and left to recover overnight at 37 °C in RPMI 1640 supplemented with 10% FBS, 1% L-glutamine and 1% penicillin/streptomycin.

### *In vitro* β cell proliferation assay

INS1 cells were cultured and maintained as previously described^46^. Cells were seeded at a density of 10^6^ in a 60 mm dish and incubated overnight at 37 °C. The following day, cells were either maintained in culture media (control) or treated with the indicated agonist for 48 hours prior to analysis by flow cytometry. After the first 24 hour treatment period, BrdU was added to culture media at a concentration of 0.1 mg/ml. Following 48 hours of agonist treatment, cells were fixed, permeabilized, and labeled with anti-BrdU antibody using a FITC BrdU Flow Kit purchased from BD Pharmingen (San Jose, CA), according to the manufacturer’s protocol. Cells were counted by flow cytometry, with a minimum of 30,000 events counted at a rate of no more than 400 events per second. Dead and apoptotic cells were excluded from counts by Annexin V and propidium iodide staining.

### *Ex vivo* β cell proliferation assay

β cell proliferation was assessed by two separate methods. First, a blinded, small-scale *ex vivo* assay was performed on intact islets isolated from WT mice as previously described^47^. Compounds were initially screened at multiple concentrations (0.1–5 mM) to assess dose-response. Subsequent experiments were carried out with acetate, 2-BA, and CPCA at the indicated concentrations, as determined by dose response curve. Placental lactogen (PL) at 0.5 ug/ml served as a positive control for β cell proliferation^38^. β cell proliferation was visualized by insulin and Ki67 immunolabeling in mildly dissociated islets. Slides were scanned using an Aperio ScanScope FL scanner and images processed as previously described^48^.

Second, proliferation was assessed by 5-bromo-2-deoxyuridine (BrdU) incorporation as previously described with slight modifications^49^. WT and *Ffar2*^−/−^ islets were cultured for 4 days with media supplemented with or without prolactin (500 ng/mL); Exendin-4 (100 nM) or acetate (1 mM). The media was changed every second day. 500 ng/mL BrdU (Sigma) was added to the culture media during the last 24 hours. Islets were dispersed with 0.05% trypsin and fixed on poly-L-lysine coated coverslips with 4% paraformaldehyde. Epitopes were retrieved by treatment with 1N hydrochloric acid for 25 minutes at 37 °C. Immunostaining of BrdU and insulin was performed on processed islets, and β-cell proliferation rate was determined by quantifying percentage of BrdU-positive insulin-positive cells. Following antibodies were used: primary-rat anti-BrdU (1:100, Abcam), guinea pig anti-insulin (1:100, Abcam); secondary-FITC goat anti-guinea pig IgG (1:200) (Vector Laboratories), AlexaFluor-594 goat anti-rat IgG (1:200, Life Technologies, Madison, WI).

### Differential methylation analysis

Differentially methylated insulin DNA was measured by multiplex PCR analysis using terminal serum collected from 4–5 animals per age and genotype as previously described^34^. Unmethylation index is the ratio of unmethylated preproinsulin DNA to methylated preproinsulin DNA in serum.

### Assessment of Ca^2+^ mobilization and cAMP accumulation by FFA2

Calcium mobilization was assessed in the βTC3 mouse β cell line, a cell line that does not express FFA3, a related receptor^28^. Cells were seeded overnight and serum starved in a 96-well black wall/clear bottom plate. On the day of the experiment, cells were loaded with 5 μM Fluo-8 (AAT Bioquest, Sunnyvale, CA) and incubated at 37 °C for 60 minutes. Compounds were next titrated at multiple concentrations and fluorescence was measured using a fluorometric imaging plate reader (FLIPR). For cAMP assays, CHO-K1 cells were transfected with an expression vector containing full-length human FFA2 cDNA (GenBank Accession Number NM_005306) with FLAG tag sequence at N-terminus, and assays performed as previously described^50^. This assay was performed at Multispan, Inc. In each experiment, each compound was tested in quadruplicate at multiple concentrations. The pE_max_ and pEC_50_ were obtained for each compound based on concentration-response curves generated using GraphPad Prism.

### RNA-seq and data analysis

Total cellular RNA was extracted from isolated islets (pooled from 2 mice) using an RNeasy Mini kit (QIAGEN, Venlo, Linburg). Experiments were run in triplicate for each genotype. RNA seq and data analyses were carried out by the Next Generation Sequencing Core, Center for Genetic Medicine, Northwestern University. Briefly, alignment and expression analysis were performed using TopHat (v2.0.8b) and Cufflinks (v2.1.1). Differential expression was determined by cuffdiff using an FDR cutoff value of 0.05. The results of the differential expression analysis were processed with the R package, cummerbund (http://compbio.mit.edu/cummeRbund/) to obtain up-and down-regulated genes.

## Additional Information

**How to cite this article**: Villa, S. R. *et al.* Loss of Free Fatty Acid Receptor 2 leads to impaired islet mass and beta cell survival. *Sci. Rep.* **6**, 28159; doi: 10.1038/srep28159 (2016).

## Supplementary Material

Supplementary Information

## Figures and Tables

**Table 1:** Signaling properties of the FFA2 agonist CPCA.

	CPCA	Acetate
Calcium EC_50_	7.8 ± 0.4	2.3 ± 0.2
	(n = 3)	(n = 13)
cAMP EC_50_	2.7 ± 0.4	4.4 ± 0.2
	(n = 4)	(n = 6)

pEC_50_ of CPCA in calcium mobilization and cAMP accumulation assays is shown. Acetate values shown for comparison. Values are mean ± SEM.
