# Supplementary material for: Loss of Free Fatty Acid Receptor 2 leads to impaired islet mass and beta cell survival
Source: Sci Rep. 2016 Jun 21;6:28159. doi: 10.1038/srep28159 (PMC4914960; doi:10.1038/srep28159)
Supplement: Supplementary Information [file srep28159-s1.doc]

**Supplement to:**

**Loss of Free Fatty Acid Receptor 2 leads to impaired islet mass and beta cell survival**

Stephanie R. Villa^1^, Medha Priyadarshini^1^, Miles H. Fuller^1^, Tanya Bhardwaj^1^, Michael R. Brodsky^1^, Anthony R. Angueira^1^, Rockann E. Mosser^2^, Bethany A. Carboneau^3^, Sarah A. Tersey^4^, Helena Mancebo^5^, Annette Gilchrist^6^, Raghavendra G. Mirmira^4,7,8^, Maureen Gannon^2,3,9^, and Brian T. Layden^1,10^

^1^Division of Endocrinology, Metabolism and Molecular Medicine, Northwestern University Feinberg School of Medicine, Chicago, IL, U.S.A.

^2^Vanderbilt University, Department of Medicine, Division of Diabetes, Endocrinology and Metabolism, Nashville, TN, U.S.A.

^3^Vanderbilt University, Department of Molecular Physiology and Biophysics, Nashville, TN, U.S.A.

^4^Department of Pediatrics and the Herman B Wells Center for Pediatric Research, Indiana University School of Medicine, Indianapolis, IN U.S.A.

^5^Multispan, Hayward, CA, U.S.A.

^6^Midwestern University Department of Pharmaceutical Sciences, Downers Grove, IL, U.S.A.

^7^Department of Biochemistry and Molecular Biology, Indiana University School of Medicine, Indianapolis, IN, U.S.A.

^8^Department of Medicine, Indiana University School of Medicine, Indiana University, Indianapolis, IN, U.S.A.

^9^Tennessee Valley Health Authority, Department of Veterans Affairs, Nashville, TN, U.S.A.

^10^Jesse Brown Veterans Affairs Medical Center, Chicago, IL, U.S.A.

**Supplementary Figure 1.** β cell proliferation in WT and *Ffar2^-/-^* mice as determined by Ki67 co-localization with insulin-positive cells. Analysis conducted at 10 weeks of age following 1 week on HFD, or at 26 weeks of age following 20 weeks on HFD.


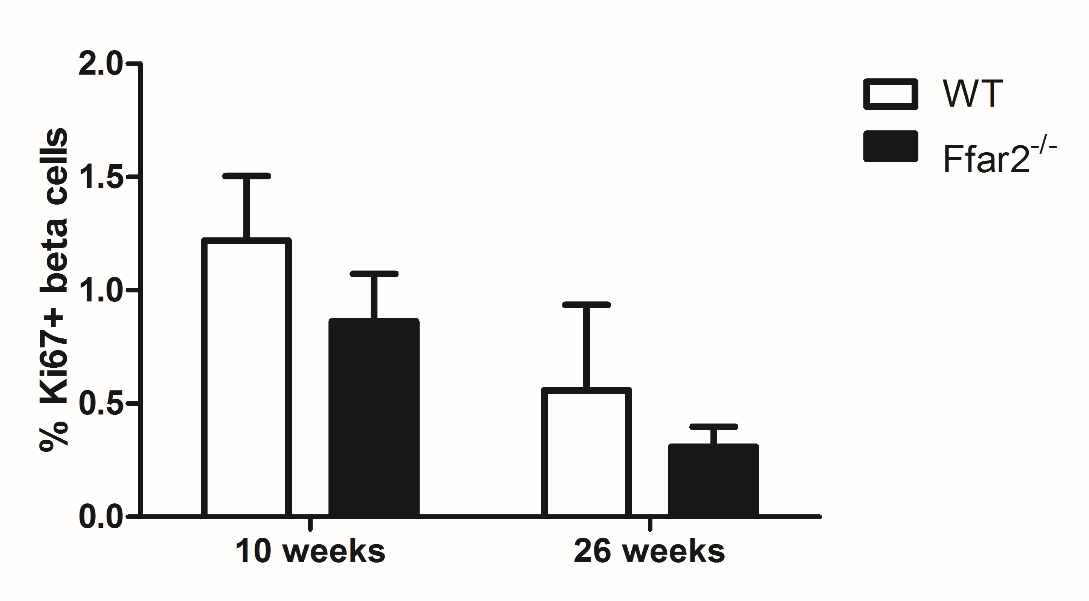


**Supplementary Table 1.** Expression of key genes in the PRLR signaling pathway in FFAR2 WT and KO islets. Islet gene expression was analyzed by RNAseq using RNA extracted from male FFAR2 WT and KO islets (n = 3 per genotype, 13 – 15 weeks old).

| **Gene Name** | **RefSeq ID** | **Log2 Fold Change** | **Test Statistic** | **FDR-adjusted p-value** |
| --- | --- | --- | --- | --- |
| **Prlr** | [**NM_001253782**](http://www.ncbi.nlm.nih.gov/nuccore/NM_001253782) | **-0.125** | **-0.314** | **0.999** |
| **Jak2** | [**NM_001048177**](http://www.ncbi.nlm.nih.gov/nuccore/NM_001048177) | **0.252** | **0.784** | **0.999** |
| **Stat5a** | [**NM_001164062**](http://www.ncbi.nlm.nih.gov/nuccore/NM_001164062) | **0.430** | **1.264** | **0.568** |
| **Stat5b** | [**NM_001113563**](http://www.ncbi.nlm.nih.gov/nuccore/NM_001113563) | **0.318** | **1.068** | **0.767** |
| **Ccnd1** | [**NR_039539**](http://www.ncbi.nlm.nih.gov/nuccore/NR_039539) | **-0.151** | **-0.454** | **0.999** |
| **E2f1** | [**NM_007891**](http://www.ncbi.nlm.nih.gov/nuccore/NM_007891) | **-0.161** | **-0.471** | **0.999** |
| **E2f2** | [**NM_177733**](http://www.ncbi.nlm.nih.gov/nuccore/NM_177733) | **-0.405** | **-0.862** | **0.999** |
| **E2f3** | [**NM_010093**](http://www.ncbi.nlm.nih.gov/nuccore/NM_010093) | **-0.086** | **-0.175** | **0.999** |
| **E2f4** | [**NM_148952**](http://www.ncbi.nlm.nih.gov/nuccore/NM_148952) | **0.149** | **0.543** | **0.999** |
| **E2f5** | [**NM_007892**](http://www.ncbi.nlm.nih.gov/nuccore/NM_007892) | **0.062** | **0.156** | **0.999** |
| **E2f6** | [**NM_033270**](http://www.ncbi.nlm.nih.gov/nuccore/NM_033270) | **0.081** | **0.254** | **0.999** |
| **Rb1** | [**NM_009029**](http://www.ncbi.nlm.nih.gov/nuccore/NM_009029) | **-0.125** | **-0.360** | **0.999** |
